# 1421. Spatial distribution of surface microbiota and resistance genes within and between rooms in an intensive care unit

**DOI:** 10.1093/ofid/ofad500.1258

**Published:** 2023-11-27

**Authors:** Matthew J Ziegler, Elizabeth Huang, Laura Cowden, Ebbing Lautenbach, Brendan Kelly

**Affiliations:** University of Pennsylvania, Philadelphia, PA; University of Pennsylvania, Philadelphia, PA; University of Pennsylvania, Philadelphia, PA; University of Pennsylvania, Philadelphia, PA; Hospital of the University of Pennsylvania, Philadelphia, Pennsylvania

## Abstract

**Background:**

Environmental transmission of bacteria, including multidrug-resistant organisms (MDROs), persists within hospitals despite routine cleaning. In prior work, we identified discordance between 16S profiles of room surfaces and culture of MDROs, suggesting unique environments for MDRO persistence within rooms. To build on this work, we used culture-enriched metagenomic sequencing to identify a broad range of viable taxa on surfaces with strain-level assignment and resistome profiling. Additionally, we evaluated the role of room proximity on sharing bacterial taxa and resistance genes between rooms.

**Methods:**

We selected three intensive care unit (ICU) rooms at the Hospital of the University of Pennsylvania with varying proximity and sampled 12 surfaces per room divided into three composite samples (Table 1). Sampling was repeated for 3 consecutive days. Samples underwent liquid media enrichment, DNA extraction and shotgun metagenomic sequencing. Strain-level assignment was performed with MetaPhlAn 4.0 and resistance genes assigned (strict and perfect alignment) via the Comprehensive Antibiotic Resistance Database (CARD).

**Table 1.**
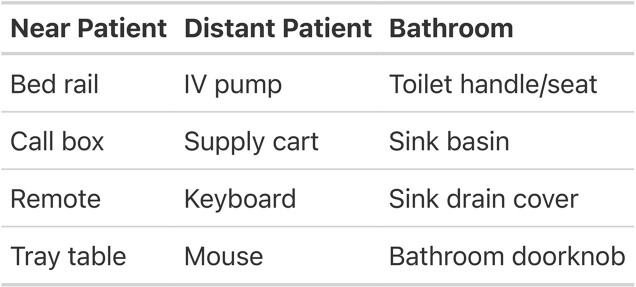
Composite surface sampling sites within intensive care unit rooms

Each composite sample represents approximately 350 square inches of surface area

**Results:**

We identified 40 strain-level assignments, with 18 shared between rooms. Within individual patient rooms, we identified microenvironments of bacterial strains and resistance genes (Figure 1 & 2) and an increased probability of detecting cephalosporin resistance genes as distance increased from the patient’s bed and neared bathroom sites (Figure 2). Using mixed effects regression, we found that distance between patient rooms was associated with decreased similarity in microbiomes of the distant in-room sites (0.9% decrease in similarity per meter increase in distance, 95% CI 0.3% to 1.5%, P=0.005), while an opposite pattern was observed in the bathroom sites (1.8% increase in similarity per meter increase in distance, 95% CI 1.2 – 2.3%, P< 0.001) (Figure 3).

**Figure 1.**
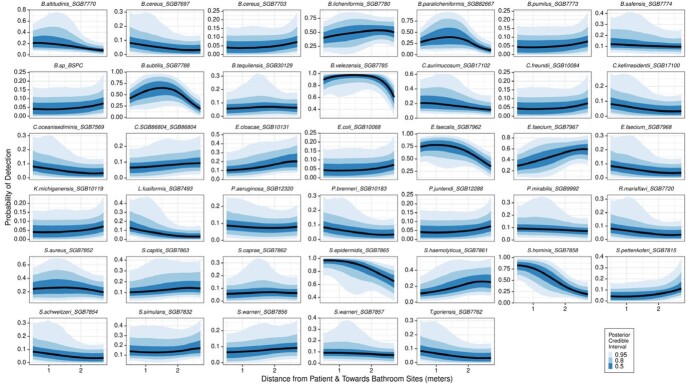
Probability of detecting bacterial strains within patient rooms as distance increases from the patient’s bed and nears the bathroom

**Figure 2.**
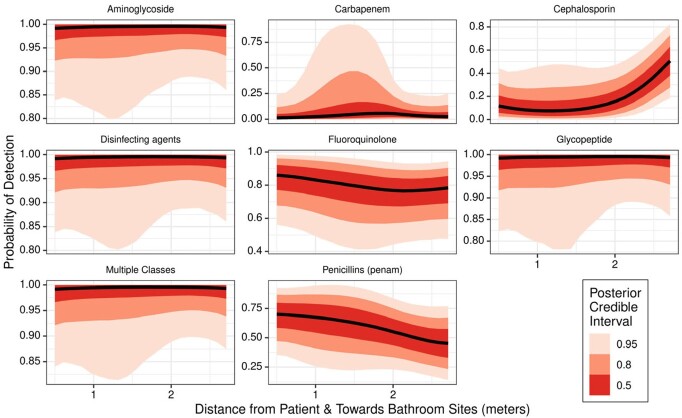
Probability of detecting bacterial resistance genes within patient rooms as distance increases from the patient’s bed and nears the bathroom. Inferred resistance by antibiotic class adapted from the Comprehensive Antibiotic Resistance Database (CARD)

**Figure 3.**
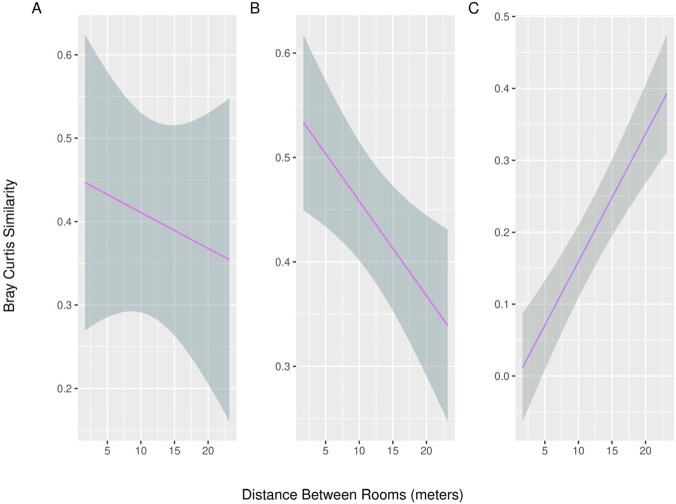
Predicted microbiome similarity versus distance between rooms sampled: A) near-patient environment B) distant-patient environment C) bathroom. Bray Curtis Similarity represents similarity of microbiome composition (0= completely dissimilar, 1 = identical composition). Grey area represents a 95% confidence interval.

**Conclusion:**

Rooms that were closer had greater similarity of microbiomes at the distant in-room site. Focused interventions on this microenvironment within patient rooms may be highest yield in reducing room-to-room transmission of bacteria. Shared microbiota at the bathroom site may be linked to plumbing infrastructure, but further research is needed to support this hypothesis.

**Disclosures:**

**All Authors**: No reported disclosures

